# Association Between Body Iron Status and Biological Aging

**DOI:** 10.3390/nu17091409

**Published:** 2025-04-23

**Authors:** Ann Von Holle, Sahana Ramamurthy, Mary V. Díaz Santana, Jacob K. Kresovich, Jack A. Taylor, Zongli Xu, Katie M. O’Brien, Dale P. Sandler, Clarice R. Weinberg

**Affiliations:** 1 Biostatistics and Computational Biology Branch, National Institute of Environmental Health Sciences, Durham, NC 27709, USA; 2 North Carolina State University, Raleigh, NC 27606, USA; 3 Department of Cancer Epidemiology, Moffit Cancer Center, Tampa, FL 33612, USA; 4 Epidemiology Branch, National Institute of Environmental Health Sciences, Durham, NC 27709, USA

**Keywords:** DNA methylation, biomarkers, ferritin, oxidative stress, telomere

## Abstract

Background/Objectives: Iron is necessary for bodily function, but abnormal levels can increase the risk of chronic diseases. Studies of leukocyte telomere length suggest blood iron levels are positively associated with cellular senescence and accelerated aging. However, associations between blood iron and more robust metrics of biological aging, specifically those based on DNA methylation, have not been examined. Methods: In a random sample of women from the Sister Study (*n* = 1260) with measured serum iron (ferritin, iron, transferrin saturation), we used linear regression models to assess cross-sectional associations between standardized serum iron and three methylation-based biological aging metrics (GrimAgeAccel, PhenoAgeAccel, and DunedinPACE), with and without adjustment for smoking, alcohol, menopause status, education, time since menopause, exercise, and diet. Results: In adjusted models, a one standard deviation increase in serum ferritin was positively associated with higher standardized levels of DunedinPACE, GrimAgeAccel, and PhenoAgeAccel (DunedinPACE: 0.05, (0.00, 0.10); PhenoAgeAccel: 0.06 (0.00, 0.11); GrimAgeAccel: 0.06 (0.01, 0.11)). In contrast, higher serum iron and transferrin saturation were inversely associated with the biological aging metrics (serum iron, DunedinPACE: −0.02, (−0.07, 0.03); PhenoAgeAccel: −0.04 (−0.10, 0.01); GrimAgeAccel: −0.05 (−0.10, −0.01); transferrin saturation (DunedinPACE: −0.01, (−0.06, 0.05); PhenoAgeAccel: −0.01 (−0.06, 0.05); GrimAgeAccel: −0.05 (−0.10, −0.01))). Conclusions: The positive association with ferritin is consistent with the proposed role of oxidative stress in accelerated aging associated with high iron exposure. However, the observed inverse associations with serum iron and transferrin saturation are not consistent with this common explanation, and future studies are needed to examine potential explanations.

## 1. Introduction

Iron is an essential element for a variety of cellular processes, including DNA repair, oxygen transfer, and enzymatic activities [1,2]. However, at high levels, iron can lead to adverse health outcomes by causing damage to cells and DNA through oxidative stress via reactive oxygen species [3,4,5]. For example, people with a hereditary iron overload syndrome (hemochromatosis) are at increased risk for a number of diseases, including liver disease, diabetes, and heart disease [6]. Serum iron, serum ferritin, and transferrin saturation are three of the most common iron status indicators in clinical settings and research studies. These measurements can be useful because they represent distinct mechanisms of iron metabolism in the body. Serum ferritin is a biomarker for iron stores, serum iron levels represent the amount of circulating iron bound to transferrin, and transferrin saturation is another measure of transport representing the percentage of iron bound to transferrin [7,8].

In a large, nationally representative study of the United States, ferritin was positively associated with accelerated biological aging, as measured by leukocyte telomere length [9]. Only two studies examined serum transferrin percentage saturation and aging, with both reporting similar associations [10,11]. One of these studies restricted their sample to people identified as having either hemochromatosis or iron overload [12], reflecting elevated baseline serum iron levels relative to a general population. These reports discussed the contribution of iron levels to pro-oxidant activities as a plausible mechanism underpinning the association between higher iron levels and accelerated biological aging.

Although there has been research on iron levels in relation to some biomarkers of biological aging, specifically leukocyte telomere length, no work to date has evaluated the association between iron exposure and DNA methylation-based biomarkers of aging. Telomere length is the most commonly used biomarker for aging as it uniformly decreases with age [13], but it has limitations such as an unclear underlying biological mechanism for aging [14,15,16]. Blood DNA methylation profiles provide another source of aging biomarkers (called “epigenetic clocks”). These clocks demonstrate clear associations with physical and cognitive functionality and are not strongly correlated with telomere length [17]. Epigenetic clocks were designed using varying procedures and are therefore proposed to detect distinct aging processes. Some of the top-performing epigenetic clocks, including PhenoAge and GrimAge, were indirectly trained on predicting all-cause mortality [18,19]. More recently, an epigenetic clock was developed to measure the rate of decline in organ-system integrity, or ‘pace of aging’ [20]. Together, these three epigenetic clocks have the potential to expand knowledge regarding associations between iron levels and biological aging.

The aim of this research is to fill gaps in our knowledge of associations between excess iron and DNA methylation-based biomarkers of the aging process. We will address this gap in the literature by examining the association between several epigenetic clocks and three common serum iron biomarkers (serum iron, transferrin saturation, and ferritin) measured in blood samples taken at baseline from a random sample of women now participating in a U.S.-based cohort. Based on findings from research on telomeres, we hypothesized that body iron levels would be positively associated biological aging as measured through methylation-based biological age metrics.

## 2. Materials and Methods

### 2.1. Sample

We used data from the Sister Study, a prospective cohort of 50,884 women aged 35–74 years at enrollment, never diagnosed with breast cancer at baseline and reporting at least one sister previously diagnosed with breast cancer [21]. We analyzed data from 1336 non-Hispanic White women enrolled in the Sister Study who had been randomly selected for inclusion in a sub-cohort [22]. The DNA processing and methylation analysis has been described elsewhere [23,24,25]. In brief, genomic DNA extracted from whole blood was analyzed on Illumina’s Infinium HumanMethylation450 BeadChip. Preprocessing and quality control of the methylation beta-value data was accomplished with the ENmix R package [26], which included correction of fluorescent dye bias and quantile normalization to address fluorescence intensity distributions between arrays and probe design bias [22,26].

### 2.2. DNA Methylation Biomarkers Analysis

DNA methylation-based aging clocks included DNA methylation PhenoAge [18] and DNA methylation GrimAge [19] measures. Publicly available scripts provided the basis for calculating these clocks, and epigenetic age acceleration for PhenoAge (PhenoAgeAccel) and GrimAge (GrimAgeAccel) was estimated by calculating residuals from linear regression models that regressed the DNA methylation-estimated age on chronological age. We also used the DunedinPACE biomarker [20], a DNA methylation biomarker of biological aging rates derived from a longitudinal study of various organ systems.

### 2.3. Serum Iron Measures Analysis

Serum iron measures included iron (mcg/dL), serum ferritin (mcg/dL), and transferrin saturation (%). The collection and processing of these measures has been described elsewhere [27]. In brief, a Roche Cobas 6000 Chemistry analyzer was used with colorimetric assays for iron and unsaturated iron binding capacity (UIBC) and a particle enhanced immunoturbidimetric assay for ferritin. Quality control and assay calibration included lab and manufacturer products. Transferrin saturation (%) was calculated as the ratio of serum iron to the sum of serum iron and unsaturated iron binding capacity, then multiplied by 100 to create a percentage. After omitting participants missing one or more methylation or serum iron measure, 1260 participants remained (Supplementary Figure S1) in the analytic sample.

### 2.4. Statistical Analysis

Descriptive statistics were reported with median and interquartile range (IQR) values for continuous variables and percentages and counts for categorical variables. We also assessed the Spearman correlation between the iron measures. To estimate associations between the methylation-based biological aging and serum iron levels as described above, we used simple linear regression models with methylation-based aging measures as outcomes and iron levels as exposures. To construct similar scales for the three continuous iron exposure variables we standardized them by subtracting the mean and dividing by the standard deviation, so that all of the iron and aging metrics had a mean of zero and a standard deviation of one. This approach allows comparisons of association strengths across analyses to be on the same scale.

Regression models were adjusted for baseline variables that we considered potential confounders: smoking status as a categorical variable with “current”, “former”, and “never” responses; educational attainment as a categorical variable with “high school or less”, “some college education or associate’s degree”, “bachelor’s degree”, or “graduate degree” responses; alcohol use as a categorical variable with “former or never” and “current” responses; body mass index as a continuous variable; time since menopause (years) for postmenopausal women as a continuous variable; premenopausal status as a categorical variable with “yes” and “no” responses; the Healthy Eating Index (HEI-2015) [28] Total Score; and hours of total physical activity per week at baseline. The variable representing time since menopause was modeled as a product with a menopause status indicator variable to account for structural zeros for women [29] who were premenopausal at the baseline measure, with the menopause status indicator retained as an additional predictor. To assess model fit, we visually examined the distribution and quantile–quantile plots of model residuals.

To evaluate evidence of nonlinear associations between serum iron measures and the biological aging outcomes, we fit linear regression models with quartiles of serum iron measures as a categorical variable with the first quartile as the referent. We also fit the same regression models with splines for the continuous iron levels and tested differences from the simple linear regression models using the change in deviance with Chi-square tests.

After examining our primary findings, we conducted post hoc analyses, which included evaluating the association between the methylation-based aging outcomes and indicator variables for anemia of chronic inflammation. These exposures were assessed based on a subset of criteria previously used by the National Health and Nutrition Examination Survey (NHANES) study of inflammatory chronic disease anemia. In that study, using data from the third NHANES cycle (1988–1994) [30], anemia of chronic inflammation was defined as a low serum iron concentration (serum iron < 60 mcg/dL) and no other evidence of iron deficiency, based on having two or three of the following criteria: (1) transferrin saturation rate < 15%, (2) serum ferritin < 12 mcg/dL, and (3) erythrocyte protoporphyrin concentration > 1.24 μM. We only used the first two of the three criteria to create a proxy indicator variable as an exposure, as the third criterion was not measured in our study. The three methylation-based outcomes remain the same as the previously described models.

A second post hoc set of analyses examined excessive transferrin saturation, an indicator defined as greater than 45% transferrin saturation, based on measures from two previous studies [11,12]. This measure was evaluated in relation to the same three methylation-based outcomes in the previously described models. A third set of post hoc analyses stratified the regression models from the primary analyses by menopausal status.

All data handling and all analyses were conducted with R version 4.2.2 [31].

## 3. Results

Of the 1260 women in the sample, median body mass index (kg/m^2^) (IQR) was 26.2 (23.0–30.5), age at baseline (IQR) was 56 years (49–62), fewer than 10% reported currently smoking, and 67% were postmenopausal (Table 1). There was a strong correlation between serum iron and transferrin saturation, with a Spearman correlation above 0.9. The relationship between ferritin and these two measures (Supplementary Figure S2) was not very strong, with a correlation below 0.35.

For PhenoAgeAccel, GrimAgeAccel, and DunedinPACE, we found a positive adjusted association with the serum ferritin iron exposure and an inverse association with serum iron (Table 2, Supplementary Figure S3). In all models, the adjusted associations were closer to the null than the unadjusted associations. The adjusted association between transferrin saturation and the DunedinPACE outcome was close to null (−0.01, 95% CI; −0.06, 0.05), and the association was stronger in the inverse direction for the GrimAgeAccel (−0.05, 95% CI: −0.10, −0.01) measure. As part of a sensitivity analysis, we removed individuals with regression residuals exceeding three standard deviations from the model and did not find a substantive change in the coefficients.

After fitting nonlinear models of the association between serum iron exposures and the methylation-based biological aging outcomes, we found evidence to support nonlinear associations for ferritin and DunedinPACE, and for PhenoAgeAccel and GrimAgeAccel with serum iron and transferrin saturation exposures (Table 2). To characterize the nonlinear fit, we evaluated the models in which the iron exposures were divided into quartiles, with the first quartile as the referent (Table 3). Although the associations did not uniformly increase or decrease across the quartile categories when compared to the first quartile, the associations with methylation-based biological outcomes remained in the same direction as found in the models with continuous iron exposures and linear associations: positive associations for serum ferritin and inverse associations for serum iron and transferrin saturation. Overall, the GrimAgeAccel outcome had stronger associations with serum iron and transferrin saturation compared to PhenoAgeAccel.

Post hoc analyses that used a proxy indicator for anemia of chronic inflammation status instead of serum iron measures demonstrated positive associations for all biological aging markers (Supplementary Table S2). Another post hoc set of analyses included examining extreme levels of transferrin saturation (≥45%) that matches analyses from prior studies of iron and telomere length. Unadjusted associations (Supplementary Table S1) from these analyses suggested little association between higher transferrin saturation levels and methylation-based biological aging, with all estimates near a null association. After adjusting these analyses for the same variables we included in the prior regression models, the association tended towards a positive association but remained near the null with at most a 0.03 standard deviation change (95% CI: −0.02, 0.08) in any of the methylation-based age outcome measures comparing transferrin saturation ≥45% to <45%. Last, when examining the primary analyses in both pre- (Supplementary Table S3) and postmenopausal groups (Supplementary Table S4), we found similar directions of association as before with less precision in the premenopausal group, as expected with a smaller sample size.

## 4. Discussion

After investigating associations between serum-based biomarkers of iron status and methylation-based measures of biological aging, we found positive associations with ferritin that supported our hypothesis of positive associations between body iron levels and biological aging. The positive associations between ferritin and the DunedinPACE and GrimAgeAccel remained after adjusting for confounders. Results for iron and transferrin saturation, inversely associated with the three biological aging outcomes, were not consistent with our hypothesis.

Iron is essential at moderate levels for human health but toxic at very high levels, and at a minimum, we expected evidence of an association between iron status and methylation-based biomarkers for biological aging when comparing the highest to the lowest quartile. We observed this pattern for ferritin in relation to GrimAgeAccel and DunedinPACE. However, the unexpected inverse associations with transferrin saturation and serum iron persisted in the fourth quartile relative to the first. One possible explanation for these findings may be a physiological mechanism underlying a reverse causality for transferrin and serum iron in which accelerated biologic aging is responsible for a reduction in those measures of iron transport, but not ferritin.

Although there are few investigations of serum iron measures and biological aging outcomes, our results support existing findings of a positive association between ferritin and another biomarker of biological aging, telomere length [9]. In a study representing the U.S.-wide population of men and women, high ferritin levels (>200 ng/mL for women and >300 ng/mL for men) were associated with shorter telomere length (beta coefficient: −0.02 (SE = 0.009, *p*-value = 0.047), after adjusting for multiple confounders. The positive association between serum ferritin and biological aging measures support the hypothesis that increased iron levels in the body induce the production of reactive oxygen species (ROS) and pro-oxidant activity [32,33], promote cell and DNA damage, and favor faster biological aging. Although evidence supports relationships between telomere length and the effects of oxidative stress [34], the mechanisms through which oxidative stress might influence methylation-based biomarkers of biological aging are not clear.

In contrast to our findings related to ferritin, the inverse associations between transferrin saturation and biological aging seem to be at odds with previous findings of inverse associations with telomere length that represent a biological aging [11,12]. In prior studies, elevated transferrin saturation (≥45%) was also associated with increased biological aging as evidenced by shorter telomere length [12]. However, that study was based on a select clinical sample of people being screened because of suspected iron overload tendencies. Also, in another study, genetically predicted iron and transferrin saturation were positively associated with epigenetic aging acceleration measures including PhenoAgeAccel and GrimAgeAccel [35]. We evaluated transferrin saturation as a continuous and categorical variable, and we did not find evidence to support positive associations with biological aging in this sample. Furthermore, the inverse associations between the serum iron and transferrin saturation and the biological aging measures in this study do not correspond to the oxidative stress hypothesis linking higher iron levels with biological aging.

Several conditions may explain the reversals in the direction of association that we found for serum iron and transferrin saturation with biological aging. Our sample, restricted to U.S. White women, may not generalize to other results based on different populations. For example, the Hemochromatosis and Iron Overload Screening (HEIRS) Study [12] included a more ethnically diverse sample from the U.S., which were about half men, and the participants were being clinically evaluated for possible iron overload. The other study mentioned above estimated associations between iron levels and biological aging in a Korean population, which was also about half male, although the direction of association for all biomarkers remained the same when limited to females [11].

Differences in types of biological aging measures across studies could also contribute to discrepancies in our findings. Telomere length and methylation-based biomarkers are physically distinct markers of biological aging and are weakly correlated [36,37]. A recent review of telomere length as a marker of biological age suggests that it represents a different aspect of the aging process given its lack of correlation with various methylation-based biomarkers of aging [16]. The mechanism of excess transport measures of iron driving more production of reactive oxygen species, hypothesized to damage cells and DNA, may apply to reduction in telomere length but not to methylation-based biological aging changes. The potential for different mechanisms underlying each type of marker may also explain the different directions of association between iron levels and methylation-based biomarkers of aging.

Other conditions may explain the association between adverse aging changes and a pattern of higher ferritin and lower circulating iron. Anemia of inflammation is one condition that may provide clues to potential mechanisms [38,39,40,41]. Increased ferritin levels paired with decreased serum iron and transferrin levels can indicate anemia of inflammation [30,41,42]. This type of anemia can follow the experience of chronic disease [30,43,44]. A potential mechanistic pathway may include biological aging as an exposure preceding chronic disease outcomes, resulting in anemia of inflammation [45,46,47]. If so, the assumed temporality of iron levels preceding epigenetic aging may be incorrectly specified and the cross-sectional design is a limitation of this study. In post hoc analyses, we found support for this alternative explanation with a positive association between a proxy marker of anemia of inflammation and biological aging, but this association was attenuated towards the null after adjusting for potential confounders (Supplementary Table S2). Future studies could leverage a longitudinal study design to better understand the sequence of events corresponding to the course of chronic disease, iron homeostasis, and biological aging.

Another limitation of this study involved our use of standardized exposures and outcomes, which allowed comparisons across iron measures and biomarker outcomes to be on the same scale but did not match the original units for the measures. Also, the estimated coefficients for those standardized predictors are small, which raises issues related to the biological significance of the estimated associations. One advantage of this study is the assessment of three serum iron exposures with methylation-based biomarkers of aging, which expands upon the work previously performed with telomere length markers of biological age. To our knowledge, this is the first study examining iron exposure and methylation-based biological aging outcomes. Replication in an independent sample can further support or refute these findings.

## 5. Conclusions

In summary, we found a positive association between serum ferritin and biological aging, which supported our hypothesis that higher serum iron levels are associated with faster biological aging. However, these associations were not consistent across the three iron exposures, and we found a consistent inverse association between the serum-based markers for iron transport, serum iron, and transferrin saturation, and the three biological aging outcomes. One explanation for different results from prior studies based on telomere length may rest with the choice of a methylation-based biological aging outcome in this study, which may represent different mechanisms or aspects of biologic aging. Replication of these findings and further investigation into potential mechanisms, such as inflammation, may explain the apparent discordance in associations with iron levels in the body and biological aging markers.

## Figures and Tables

**Table 1:** Descriptive statistics for the sample of non-Hispanic White women.

Characteristic	*N* = 1260 ^1^
Age at menopause (years) ^2^	51 (47, 55)
Smoking status	
Current smoker	96 (7.6%)
Never smoked	662 (53%)
Former smoker	502 (40%)
Highest education completed	
Graduate degree	296 (23%)
Bachelor’s degree	330 (26%)
Some college or Associate’s degree	425 (34%)
HS or less	209 (17%)
Alcohol use	
Current	1062 (84%)
Former or never	197 (16%)
BMI, kg/m^2^	26.2 (23.0, 30.5)
Time since menopause (years) ^2^	9 (5, 15)
Postmenopausal (yes)	849 (67%)
Age at enrollment (years)	56 (49, 62)
Ferritin, ug/L	66 (36, 107)
Transferrin Saturation, %	29 (23, 36)
Iron, ug/dL	95 (75, 118)
PhenoAgeAccel	−0.5 (−4.6, 3.4)
GrimAgeAccel	−0.5 (−2.1, 1.5)
DunedinPACE	1.04 (0.99, 1.10)
Physical activity at baseline (hours per week)	13 (8, 20)
Healthy Eating Index (HEI-2015) Total Score	73 (65, 79)

^1^ Median (IQR); *n* (%). ^2^ Restricted to postmenopausal group (*n* = 849). Note: Percentages may not sum to 100 due to rounding.

**Table 2:** Linear association regression coefficients (95% confidence intervals) between serum iron levels and biological aging outcome.

Exposure	Aging Outcome	Unadjusted	Adjusted ^1^	*p*-Value, Spline Model vs. Linear Model ^2,3^
Ferritin (*n* = 1249)	GrimAgeAccel	0.11 (0.05, 0.16)	0.06 (0.01, 0.11)	0.59
	PhenoAgeAccel	0.07 (0.01, 0.12)	0.06 (0.00, 0.11)	0.24
	DunedinPACE	0.11 (0.06, 0.17)	0.05 (0.00, 0.10)	0.02
Iron (*n* = 1251)	GrimAgeAccel	−0.12 (−0.18, −0.07)	−0.05 (−0.10, −0.01)	0.04
	PhenoAgeAccel	−0.08 (−0.14, −0.03)	−0.04 (−0.10, 0.01)	0.00
	DunedinPACE	−0.09 (−0.14, −0.03)	−0.02 (−0.07, 0.03)	0.20
Transferrin saturation (%) (*n* = 1201)	GrimAgeAccel	−0.12 (−0.18, −0.06)	−0.05 (−0.10, −0.01)	0.01
	PhenoAgeAccel	−0.05 (−0.11, 0.01)	−0.01 (−0.06, 0.05)	0.00
	DunedinPACE	−0.07 (−0.12, −0.01)	−0.01 (−0.06, 0.05)	0.13

^1^ Adjusted for smoking status as a categorical variable with “current”, “former”, and “never” responses; educational attainment as a categorical variable with “high school or less”, “some college education or associate’s degree”, “bachelor’s degree”, or “graduate degree” responses; alcohol use as a categorical variable with “former or never” and “current” responses; body mass index as a continuous variable; time since menopause (years) as a continuous variable and premenopausal status as a categorical variable with “yes” and “no” responses; Healthy Eating Index (HEI-2015) Total Score; and hours of total physical activity per week at baseline. ^2^ Test of differences between spline and simple linear regression models using the change in deviance with Chi-square tests. ^3^ A *p*-value < 0.05 suggests that the spline model offers better fit relative to the linear model.

**Table 3:** Regression coefficients (95% confidence intervals) for biological aging regressed on iron quartiles.

		Unadjusted					Adjusted ^1^				
Exposure	Aging Outcome	1st Quartile	2nd Quartile	3rd Quartile	4th Quartile	* p * -Value Linear Trend	1st Quartile	2nd Quartile	3rd Quartile	4th Quartile	*p*-Value Linear Trend ^2^
Ferritin (*n* = 1210)	GrimAgeAccel	Ref	0.01 (−0.15, 0.16)	0.02 (−0.14, 0.18)	0.17 (0.02, 0.33)	0.03	Ref	0.06 (−0.07, 0.19)	−0.07 (−0.20, 0.06)	0.07 (−0.07, 0.21)	0.73
	PhenoAgeAccel	Ref	0.05 (−0.11, 0.20)	0.05 (−0.10, 0.21)	0.09 (−0.07, 0.25)	0.28	Ref	0.09 (−0.06, 0.25)	0.07 (−0.09, 0.24)	0.09 (−0.08, 0.25)	0.37
	DunedinPACE	Ref	0.15 (0.00, 0.31)	0.20 (0.04, 0.36)	0.30 (0.14, 0.46)	0	Ref	0.20 (0.05, 0.35)	0.13 (−0.02, 0.29)	0.18 (0.03, 0.33)	0.06
Transferrin saturation (*n* = 1164)	GrimAgeAccel	Ref	−0.23 (−0.39, −0.08)	−0.41 (−0.57, −0.25)	−0.41 (−0.57, −0.25)	0	Ref	−0.16 (−0.30, −0.03)	−0.22 (−0.36, −0.09)	−0.24 (−0.37, −0.11)	0
	PhenoAgeAccel	Ref	−0.09 (−0.24, 0.07)	−0.23 (−0.39, −0.07)	−0.13 (−0.29, 0.03)	0.03	Ref	−0.02 (−0.18, 0.14)	−0.14 (−0.30, 0.03)	−0.01 (−0.18, 0.15)	0.56
	DunedinPACE	Ref	−0.17 (−0.33, −0.01)	−0.24 (−0.40, −0.09)	−0.27 (−0.43, −0.12)	0	Ref	−0.12 (−0.27, 0.03)	−0.12 (−0.27, 0.03)	−0.11 (−0.26, 0.04)	0.18
Iron (*n* = 1212)	GrimAgeAccel	Ref	−0.21 (−0.36, −0.05)	−0.36 (−0.51, −0.20)	−0.39 (−0.55, −0.24)	0	Ref	−0.18 (−0.31, −0.05)	−0.25 (−0.38, −0.12)	−0.19 (−0.32, −0.06)	0
	PhenoAgeAccel	Ref	−0.23 (−0.38, −0.07)	−0.25 (−0.41, −0.09)	−0.21 (−0.37, −0.06)	0.01	Ref	−0.22 (−0.37, −0.06)	−0.19 (−0.35, −0.03)	−0.12 (−0.28, 0.04)	0.2
	DunedinPACE	Ref	−0.19 (−0.34, −0.03)	−0.34 (−0.49, −0.18)	−0.30 (−0.46, −0.15)	0	Ref	−0.18 (−0.33, −0.04)	−0.25 (−0.40, −0.10)	−0.13 (−0.28, 0.01)	0.05

^1^ Adjusted for smoking status as a categorical variable with “current”, “former”, and “never” responses; educational attainment as a categorical variable with “high school or less” “some college education or associate’s degree”, “bachelor’s degree”, or “graduate degree” responses; alcohol use as a categorical variable with “former or never” and “current” responses; body mass index as a continuous variable; time since menopause (years) as a continuous variable and premenopausal status as a categorical variable with “yes” and “no” responses; Healthy Eating Index (HEI-2015) Total Score; and hours of total physical activity per week at baseline. ^2^ Linear trend measured by transforming quartiles into corresponding numeric values from 1 to 4.

## Data Availability

Data used for this study is available upon reasonable request, as noted on the study web site (https://sisterstudy.niehs.nih.gov/English/data-requests.htm, accessed on 12 December 2024).

## Supplementary Materials

The following supporting information can be downloaded at: https://www.mdpi.com/article/10.3390/nu17091409/s1, Figure S1. Sample size flow diagram; Figure S2. Spearman correlation between serum iron measures. Figure S3. Regression coefficients for association between iron levels quartiles and biological aging measure; Table S1. Associations between anemia of chronic inflammation (ACI) and methylation-based aging outcomes; Table S2. Associations between high transferrin saturation levels and biological aging outcomes; Table S3. Linear association between serum iron levels and biological aging outcome for premenopausal group (*n* = 410); Table S4. Linear association between serum iron levels and biological aging outcome for postmenopausal group (*n* = 849).

## Abbreviations

The following abbreviations are used in this manuscript:

BMI	Body Mass Index
PhenoAgeAccel	epigenetic age acceleration for PhenoAge
GrimAgeAccel	epigenetic age acceleration for GrimAge
IQR	interquartile range
