# Supplementary material for: Association Between Body Iron Status and Biological Aging
Source: Nutrients. 2025 Apr 23;17(9):1409. doi: 10.3390/nu17091409 (PMC12073140; doi:10.3390/nu17091409)
Supplement: Supplementary file 1 [file nutrients-17-01409-s001.zip › supplement-20241205.docx]

# Supplemental material

**Supplementary Table 1. Associations between anemia of chronic inflammation (ACI) and methylation-based aging outcomes**

| **Exposure** | **Aging outcome** | **Unadjusted** | **Adjusted** |
| --- | --- | --- | --- |
| ACI (yes/no) | GrimAgeAccel | 0.07 ( 0.01, 0.12) | 0.03 (-0.01, 0.08) |
| ACI (yes/no) | PhenoAgeAccel | 0.06 ( 0.00, 0.11) | 0.05 (0.00, 0.11) |
| ACI (yes/no) | DunedinPACE | 0.04 (-0.02, 0.09) | 0.02 (-0.03, 0.08) |

**Supplementary Table 2. Associations between high transferrin saturation levels and biological aging outcomes**

| **Exposure** | **Aging outcome** | **Unadjusted** | **Adjusted** |
| --- | --- | --- | --- |
| Transferrin saturation ≥ 45% | GrimAgeAccel | -0.01 (-0.06, 0.05) | 0.03 (-0.02, 0.08) |
| Transferrin saturation ≥ 45% | PhenoAgeAccel | <0.01 (-0.05, 0.06) | 0.02 (-0.04, 0.08) |
| Transferrin saturation ≥ 45% | DunedinPACE | <0.01 (-0.06, 0.05) | 0.03 (-0.02, 0.09) |

**Supplementary Table 3. Linear association between serum iron levels and biological aging outcome for premenopausal group (n=410)**

| **Exposure** | **Aging outcome** | **Unadjusted** | **Adjusted^1^** | **p-value, test spline vs linear^2^** |
| --- | --- | --- | --- | --- |
| Ferritin | GrimAgeAccel | 0.17 ( 0.07, 0.27) | 0.10 ( 0.02, 0.19) | 0.89 |
|  | PhenoAgeAccel | 0.12 ( 0.02, 0.21) | 0.09 (-0.01, 0.18) | 0.65 |
|  | DunedinPACE | 0.17 ( 0.08, 0.27) | 0.12 ( 0.03, 0.21) | 0.27 |
| Iron | GrimAgeAccel | -0.14 (-0.24, -0.05) | -0.06 (-0.14, 0.02) | 0.10 |
|  | PhenoAgeAccel | -0.06 (-0.16, 0.04) | -0.03 (-0.13, 0.07) | 0.03 |
|  | DunedinPACE | -0.05 (-0.15, 0.04) | 0.03 (-0.07, 0.12) | 0.26 |
| Transferrin saturation | GrimAgeAccel | -0.13 (-0.23, -0.03) | -0.07 (-0.16, 0.02) | 0.20 |
|  | PhenoAgeAccel | -0.04 (-0.14, 0.06) | 0.00 (-0.10, 0.10) | 0.01 |
|  | DunedinPACE | -0.05 (-0.15, 0.05) | 0.01 (-0.08, 0.11) | 0.97 |

^1^Adjusted for smoking status as a categorical variable with “current”, “former”, and “never” responses, educational attainment as a categorical variable with “high school or less”, “some college education or associate’s degree”, “bachelor’s degree”, or “graduate degree” responses, alcohol use as a categorical variable with “former or never” and “current” responses, body mass index as a continuous variable, time since menopause (years) as a continuous variable, and premenopausal status as a categorical variable with “yes” and “no” responses, Healthy Eating Index (HEI-2015) Total Score, and hours of total physical activity per week at baseline.

^2^ Test of differences between spline and simple linear regression models using the change in deviance with Chi-square tests.

**Supplementary Table 4. Linear association between serum iron levels and biological aging outcome for postmenopausal group (n=849)**

| **Exposure** | **Aging outcome** | **Unadjusted** | **Adjusted^1^** | **p-value, test spline vs linear^2^** |
| --- | --- | --- | --- | --- |
| Ferritin | GrimAgeAccel | 0.12 ( 0.05, 0.18) | 0.06 ( 0.01, 0.12) | 0.38 |
|  | PhenoAgeAccel | 0.07 ( 0.00, 0.14) | 0.04 (-0.02, 0.11) | 0.09 |
|  | DunedinPACE | 0.09 ( 0.02, 0.16) | 0.04 (-0.02, 0.11) | 0.04 |
| Iron | GrimAgeAccel | -0.12 (-0.18, -0.05) | -0.06 (-0.12, -0.01) | 0.31 |
|  | PhenoAgeAccel | -0.09 (-0.16, -0.03) | -0.06 (-0.13, 0.00) | 0.15 |
|  | DunedinPACE | -0.10 (-0.17, -0.04) | -0.06 (-0.12, 0.00) | 0.45 |
| Transferrin saturation | GrimAgeAccel | -0.12 (-0.19, -0.05) | -0.05 (-0.10, 0.01) | 0.01 |
|  | PhenoAgeAccel | -0.06 (-0.13, 0.01) | -0.02 (-0.09, 0.05) | 0.06 |
|  | DunedinPACE | -0.08 (-0.14, -0.01) | -0.02 (-0.08, 0.05) | 0.04 |

^1^Adjusted for smoking status as a categorical variable with “current”, “former”, and “never” responses, educational attainment as a categorical variable with “high school or less”, “some college education or associate’s degree”, “bachelor’s degree”, or “graduate degree” responses, alcohol use as a categorical variable with “former or never” and “current” responses, body mass index as a continuous variable, time since menopause (years) as a continuous variable, and premenopausal status as a categorical variable with “yes” and “no” responses, Healthy Eating Index (HEI-2015) Total Score, and hours of total physical activity per week at baseline.

^2^ Test of differences between spline and simple linear regression models using the change in deviance with Chi-square tests.

**Figure captions:**

Supplementary Figure 1. Sample size flow diagram

Supplementary Figure 2. Spearman correlation between serum iron measures.

Supplementary Figure 3. Regression coefficients for association between iron levels quartiles and biological aging measure.
