# Supplementary figures and images for: Association Between Body Iron Status and Biological Aging
Source: Nutrients. 2025 Apr 23;17(9):1409. doi: 10.3390/nu17091409 (PMC12073140; doi:10.3390/nu17091409)

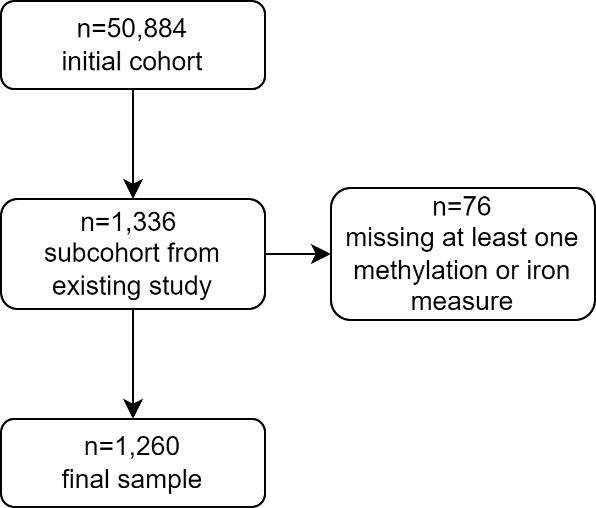

Supplement: Supplementary file 1 [file nutrients-17-01409-s001.zip › suppfig1.tif]

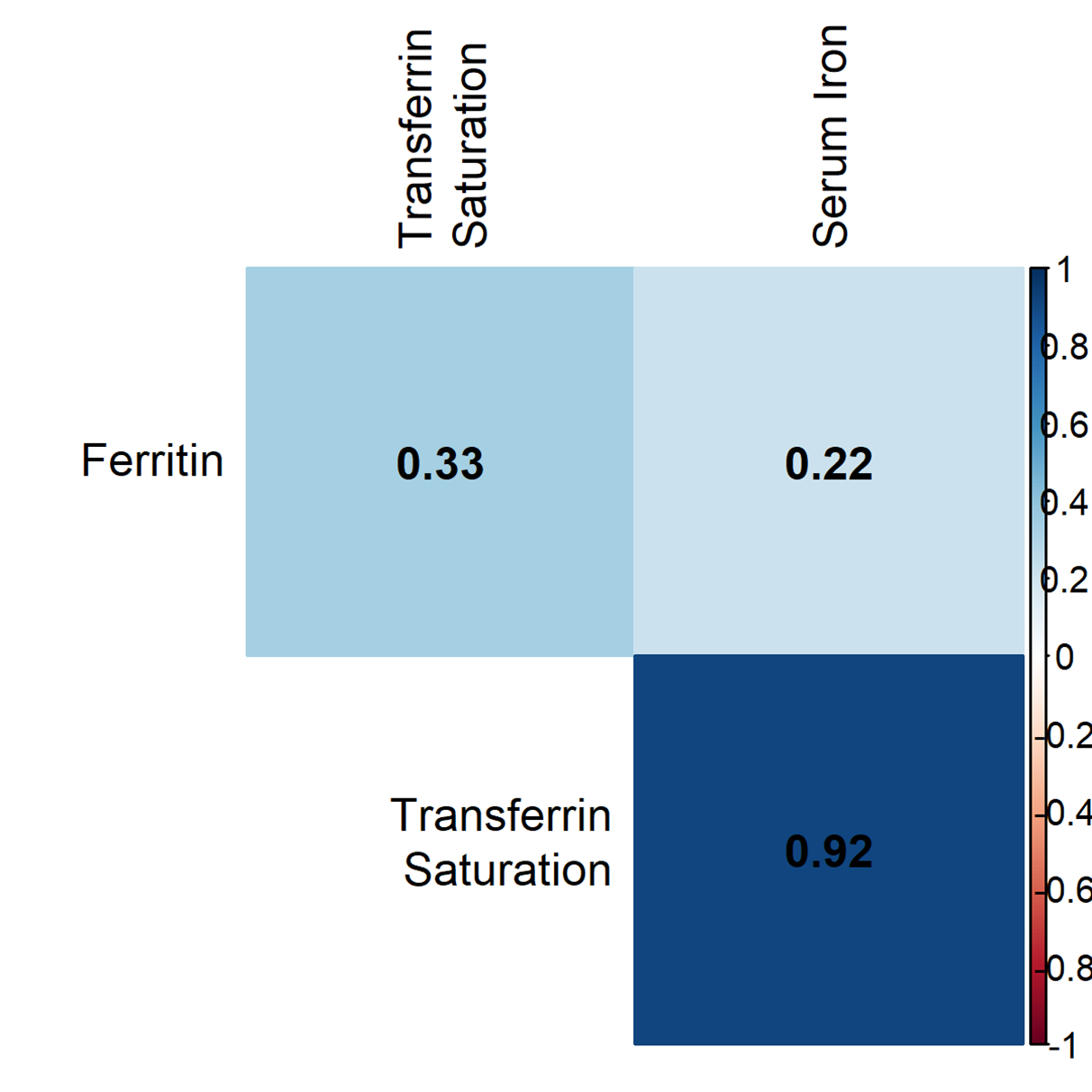

Supplement: Supplementary file 1 [file nutrients-17-01409-s001.zip › suppfig2.tif]

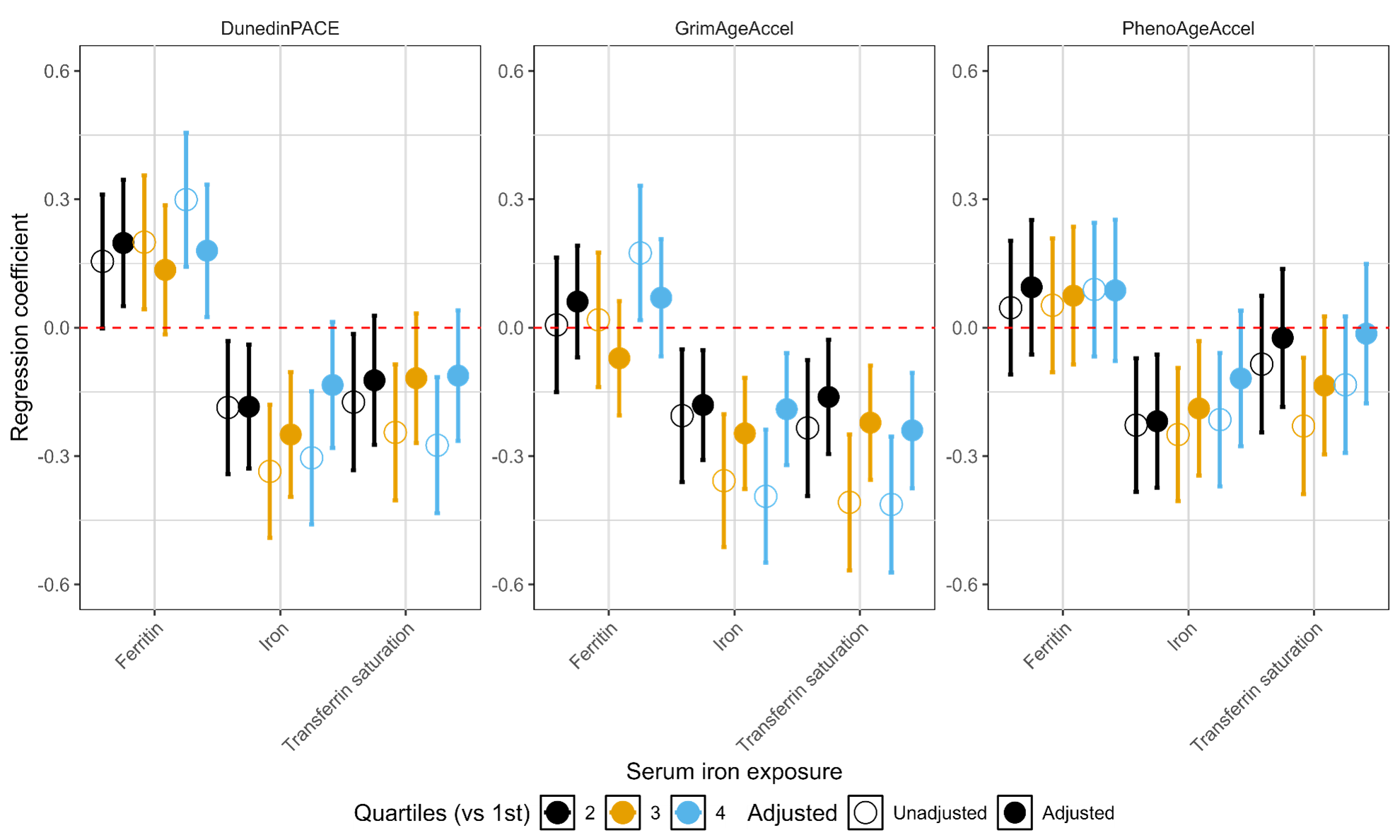

Supplement: Supplementary file 1 [file nutrients-17-01409-s001.zip › suppfig3.tif]
